# Retraction: Design and validation of ultra-compact metamaterial-based biosensor for non-invasive cervical cancer diagnosis in terahertz regime

**DOI:** 10.1371/journal.pone.0343674

**Published:** 2026-02-25

**Authors:** The PLOS One Editors

The *PLOS One* Editors retract this article [1] due to concerns about:

- Compliance with PLOS policies on Manipulation of the Publication Process, Competing Interests, and Authorship;
- Compromised peer review.

We regret that the issues were not identified prior to the publication of [1].

MNH, MTI, SL, and MSI did not agree with the retraction. IuD, BS, and SK either did not respond directly or could not be reached.
